# Treatment Strategy for Dyslipidemia in Cardiovascular Disease Prevention: Focus on Old and New Drugs

**DOI:** 10.3390/pharmacy6010010

**Published:** 2018-01-21

**Authors:** Donatella Zodda, Rosario Giammona, Silvia Schifilliti

**Affiliations:** 1 Drug Department of Local Health Unit (ASP), Viale Giostra, 98168 Messina, Italy; 2 Clinical Pharmacy Fellowship, University of Messina, Viale Annunziata, 98168 Messina, Italy; ros.giammona@gmail.com (R.G.); silvia.schifilliti@hotmail.it (S.S.)

**Keywords:** lipid lowering therapy, dyslipidemia, statins, fibrate, PCSK9 inhibitors, lomitapide

## Abstract

Prevention and treatment of dyslipidemia should be considered as an integral part of individual cardiovascular prevention interventions, which should be addressed primarily to those at higher risk who benefit most. To date, statins remain the first-choice therapy, as they have been shown to reduce the risk of major vascular events by lowering low-density lipoprotein cholesterol (LDL-C). However, due to adherence to statin therapy or statin resistance, many patients do not reach LDL-C target levels. Ezetimibe, fibrates, and nicotinic acid represent the second-choice drugs to be used in combination with statins if lipid targets cannot be reached. In addition, anti-PCSK9 drugs (evolocumab and alirocumab) provide an effective solution for patients with familial hypercholesterolemia (FH) and statin intolerance at very high cardiovascular risk. Recently, studies demonstrated the effects of two novel lipid-lowering agents (lomitapide and mipomersen) for the management of homozygous FH by decreasing LDL-C values and reducing cardiovascular events. However, the costs for these new therapies made the cost–effectiveness debate more complicated.

## 1. Introduction

Cholesterol is a fatty substance necessary for the proper functioning of the body. In fact, it participates in the synthesis of some hormones and vitamin D, and it is a constituent of cell membranes. Cholesterol is produced by the liver but can also be introduced with the diet (foods rich in animal fats such as meat, butter, salami, cheese, egg yolk, and liver) [1].

Cholesterol is transported through the blood thanks to a particular class of particles called lipoproteins. There are four types of lipoprotein classified by density, which is inversely proportional to the amount of cholesterol present [1]. The most important for cardiovascular prevention are: low-density lipoproteins (LDL), carrying liver cholesterol to the cells, and high-density lipoproteins (HDL) that remove excess cholesterol from different tissues and carry it back to the liver, which then eliminates it [2].

LDLs are known in the common language as “bad cholesterol” because when they are present in excessive amounts, they tend to settle on the walls of arteries, causing thickening and progressive hardening [3]. This process, called atherosclerosis, can lead to the formation of true plaques (or atheromas) thus altering blood flow, or even block it completely. When the heart does not get enough oxygen-rich blood, angina pectoris may develop—a condition characterized by chest pain, which can radiate to the arms or jaw, usually at the same time as effort or stress. In addition, the plaques may detach and form a thrombus, which can cause a sudden stopping of the bloodstream [4]. Depending on where it is located, obstruction of a vessel may cause myocardial infarction, stroke, or intermittent claudication (at the lower limb level) [4].

For all these reasons, with dyslipidemia, it is important to identify a “desirable” cholesterol value that should be reached or not exceeded in order to maintain cardiovascular risk within acceptable limits. These values have been set by the various US [5] and European [6] guidelines and recommendations issued over time, even if they are not uniform for all subjects and/or patients as they depend on the clinical characteristics of the individual (primary prevention, presence of comorbidities such as diabetes, hypertension, etc.) and the presence of other risk factors (familiarity, habitual smoking, sedentary lifestyle, etc.).

High cholesterol levels do not produce direct symptoms: many people ignore the fact that they suffer from hypercholesterolemia. However, cholesterol can be easily measured with a simple blood test and must be kept under constant control. We talk about hypercholesterolemia when total cholesterol (LDL plus VLDL and HDL) is too high.

However, generally, desirable cholesterol levels are up to 200 mg/dl for total cholesterol, up to 100 mg/dl for LDL cholesterol (LDL-C), and not less than 50 mg/dl for HDL cholesterol (HDL-C). When plasma cholesterol concentrations exceed these levels, it is referred to as hypercholesterolemia. 

The benefits of hypercholesterolemia treatment have been confirmed by population registers in the real world [7], as well as subsequent meta-analyses of numerous randomized controlled trials (RCTs) on over 170,000 patients by the Cholesterol Treatment Trialists’ Collaboration [8,9], pointing out that a reduction of 1 mmol/L (approximately 38 mg/dl) of LDL-C is associated with a 20–25% reduction in the relative risk of new major cardiovascular events (cardiovascular mortality and non-fatal infarction).

Prevention and treatment of dyslipidemia should therefore be considered as an integral part of individual cardiovascular prevention interventions, which should be addressed primarily to those at higher risk who will benefit most. The most commonly used options for the pharmacologic treatment of dyslipidemia are statins, resins, fibrate, niacin, and their combinations. However, other possibilities in selected cases are available, such as PCSK9 inhibitors and the new, approved selective inhibitor of microsomal triglyceride transfer protein and antisense oligonucleotide drug.

A PubMed/Medline systematic search was performed using the key phrases “management of hypercholesterolemia”, “guidelines for management of hypercholesterolemia”, and “pharmacological management of hypercholesterolemia.” The time period of the literature search was limited to the past 23 years (1994–2017 inclusive) to capture the most recent literature in this field. All abstracts were screened and relevant articles were selected. All the most significant studies on the pharmacological management of hypercholesterolemia were selected.

## 2. Lipid-Lowering Drugs

### 2.1. Statins

**Figure 1:**
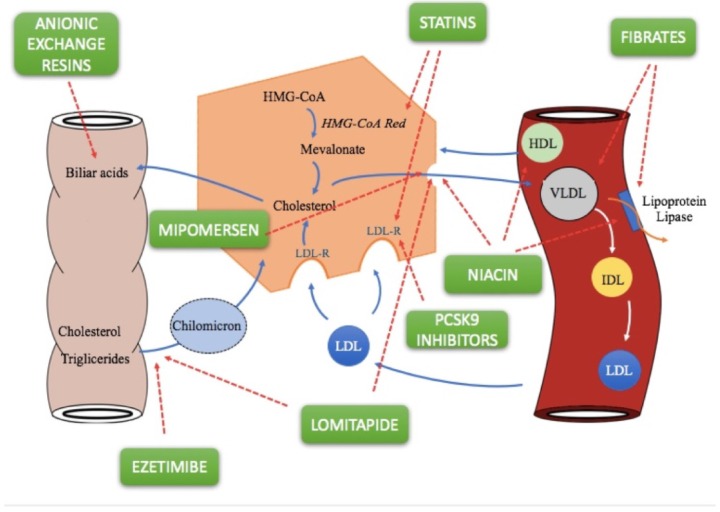
Cholesterol and triglyceride metabolism, and molecular mechanisms of lipid-lowering drugs [12,18].

Statins lower cholesterol levels through three mechanisms linked to each other [10]. The first is the selective and competitive inhibition of 3-hydroxy-3-methylglutaryl-coenzyme A (HMG-CoA) reductase, an enzyme that limits the conversion speed of HMG-CoA to mevalonic acid, a precursor of sterols, including cholesterol. Inhibition of this enzyme initially leads to a reduction in liver cholesterol, but compensatory mechanisms induce greater expression of both HMG-CoA reductase and LDL receptors (Figure 1) [10]. In the latter case, statins therefore act with an indirect mechanism, by increasing receptor-mediated absorption of LDL, hence reducing plasma LDL. Thanks to the higher number of receptors, they also reduce VLDL and IDL, which are LDL precursors: this third mechanism of action further contributes to lowering plasma LDL-C. In particular, atorvastatin and rosuvastatin produce a marked decrease in plasma triglycerides (TG), as they remove larger amounts of VLDL rich in triglycerides [11]. Statins are structural analogues of the HMG-CoA intermediate, which is formed by HMG-CoA reductase in mevalonate synthesis. Only lovastatin and simvastatin are inactive lattons, which are hydrolysed in vivo into the corresponding active β-hydroxy acid form [12].

All statins possess very low systemic bioavailability due to an extensive first-pass effect. Lovastatin and simvastatin, unlike most statins, are administered as inactive lactone prodrugs [13]. Statins differ mainly in the degree of metabolism and the number of active and inactive metabolites. All statins have active metabolites so that their activity depends also on the profile of both parent compound and active metabolites. Pravastatin has the lowest protein-binding (around 50%) when compared to other statins (>90%); furthermore, statins have a low half-life (1–4 h), while atorvastatin and rosuvastatin possess the longest terminal half-life (11–20 h) [13].

All statins are indicated in cases of primary hypercholesterolemia and mixed dyslipidemia in patients who do not respond to diet, exercise, and other non-pharmacological methods. While fluvastatin, lovastatin, and pravastatin do not lower LDL levels in homozygous familial hypercholesterolemic patients unable to produce LDL receptors, atorvastatin, rosuvastatin, and simvastatin, instead, are effective in this disease, probably due to their ability to produce a significant decrease in liver production of LDL cholesterol [6,14]. All statins can be used in cardiovascular prevention as adjuvants to reduce other risk factors with other cardioprotective therapies [15]. In the case of hypercholesterolemia, the recommended dose is 10–20 mg/day administered in a single dose in the evening; patients requiring a large reduction in LDL-C (greater than 45%) may start with 20–40 mg/day administered in a single dose in the evening [11]. Only rosuvastatin should be initiated with a dosage of 5–10 mg/day, reaching maximum doses of up to 40 mg/day only in patients who have not reached the therapeutic goals established with the lowest doses [11]. In the case of homozygous familial hypercholesterolemia, the recommended dose is 40 mg/day in the evening. In the case of cardiovascular prevention, the usual dose ranges from 20 to 40 mg/day administered in single dose at night, while for atorvastatin, a dose of 10 mg/day is used, although it may be increased as needed [15].

However, we have to mention that important pharmacokinetic interactions can affect the activity/toxicity of statins [16]. Azole antifungins, grapefruit juice, erythromycin, and clarithromycin all are potent inhibitors of cytochrome CYP3A4, therefore increasing the plasma levels of atorvastatin, lovastatin, and simvastatin, and may result in increased risk of severe myopathy or rhabdomyolysis [16]. The combination of statins with fusidic acid, fibrates, niacin, or cyclosporine may cause a greater risk of severe myopathy or rhabdomyolysis. If they are associated with bile acid sequestrants, they should be taken at least one hour before or 4–6 h later, otherwise a lower bioavailability of HMG-CoA reductase inhibitors (HMGRIs) may occur. The interaction between spironolactones and statins can lead to additive effects of decreasing concentration and activity of endogenous steroid hormones. Cimetidine, ranitidine, and omeprazole may increase blood levels of fluvastatin, while rifampicin causes more rapid elimination [17]. Coadministration of antacids results in a reduction in atorvastatin and rosuvastatin levels, but no effect on plasma LDL reduction; rosuvastatin should be given at least 2 h after an antacid. HIV protease inhibitors and diltiazem taken together with atorvastatin, lovastatin, or simvastatin lead to increased risk of myopathy, as with lovastatin and amiodarone or danazol. The anticoagulant activity of warfarin may increase if administered with fluvastatin, lovastatin, rosuvastatin, or simvastatin [17].

### 2.2. Fibrates

Fibrates (fenofibrate, bezafibrate, ciprofibrate, and gemfibrozil) are a class of lipid-lowering drugs and exert their effects mainly by activating the peroxisome proliferator-activated receptor-alpha (PPAR-alpha), while bezafibrate is an agonist for all three PPAR isoforms (alpha, gamma and delta). Fibrates decrease triglyceride levels and increase HDL-C levels (Figure 1); the latter effect is more pronounced in patients with hypertriglyceridemia [19]. The effect on LDL-C levels varies. They may reduce LDL-C levels in patients with low triglycerides, but may paradoxically increase levels for patients with high triglyceride levels [20], and significantly reduce the levels of highly atherogenic remnant lipoproteins in a more efficient way than statins.

The recommended dose for adults of Bezafibrate is 200 mg three times a day, or 400 mg of modified release tablet a day at the main meals [21]. Fenofibrate can be administered once a day (200 mg) or with four 67 mg capsules, if required. However, there are some fenofibrate formulations using a NanoCrystal technology (48 and 145 mg) that eliminates the requirement of taking the drug with a meal, or micronized capsules (67, 134, and 200 mg) resulting in greater solubility and improved bioavailability [22]. The Gemfribozil dose range is 900–1200 mg daily.

Fibrates have near 100% oral bioavailability, although fenofibrate, in its immediate release form, has only 60% oral bioavailability. Clofibrate and fenofibrate differ from other drugs of the same class since they are given as a prodrug [23]. Fenofibric acid is the circulating pharmacologically-active moiety in plasma after oral administration of fenofibrate—the ester of fenofibric acid. The absolute bioavailability of fenofibric acid is approximately 81%. Fenofibric acid is primarily conjugated with glucuronic acid and then excreted in urine [24]. In vivo data on fenofibrate metabolism reported that fenofibric acid does not undergo oxidative metabolism, e.g, by cytochrome (CYP) P450, to a significant extent [24].

The Bezafibrate Infarction Prevention study suggested that bezafibrate prevented cardiovascular events in the subgroup of coronary artery disease patients with high triglycerides [25]. Moreover, further analyses showed that bezafibrate significantly reduced new-onset diabetes [26] and myocardial infarction in the patients with metabolic syndrome [27]. These beneficial clinical effects might be explained, not only by its triglyceride-lowering effect, but also its various PPARα-mediated pleiotropic effects. Fenofibrate as monotherapy decreases serum TG levels by 20–50% and increases HDL-C levels by 10–50% [28]. The rise in HDL-C levels depends on baseline HDL-C concentrations, with the greatest elevations observed when baseline HDL-C is <39 mg/dL [29].

A Cochrane Review and meta-analysis (six eligible trials including 16,135 individuals), which compared fibrate therapy to placebo or usual care, found moderate-quality evidence for a reduced risk of CVD events (CVD death, non-fatal myocardial infarction, or non-fatal strokes) of 16%, and for coronary events (coronary heart disease death or non-fatal myocardial infarction) of 21% in trials of primary prevention of cardiovascular disease [30].

Very early in the use of clofibrate, a high incidence of myopathy was described [23]. The statement that the combination of gemfibrozil and lovastatin can lead to myopathy was made within a few months of lovastatin becoming available for prescription use [31]. This interaction between gemfibrozil and statins became tragically clear after cerivastatin became commercially available [32]. Additional studies established that higher plasma statin concentrations were due, in part, to the competition between gemfibrozil and statins for phase II enzymes that glucuronidate the statin drugs (except for fluvastatin) leading to reduced statin clearance. Higher plasma concentrations of cerivastatin were commonly toxic to muscle. However, this effect seems to be much less of a problem with other fibrates, as they undergo conjugation by using different enzymes [33].

### 2.3. Bile Acid Sequestrants

Bile acid-binding resins, including cholestyramine, colesevelam, and colestipol, are orally administered anion-exchange resins that are neither absorbed systemically nor metabolised by digestive enzymes. At the intestinal level, therefore, they bind to the two main biliary acids (glycocholic acid and taurocholic acid) making an insoluble complex that is excreted with the faeces (Figure 1) [34]. This leads to a continuous, though partial, removal of bile acids from the enterohepatic circulation. Consequently, the lower concentration of these in the liver obstructs 7α-hydroxylase feedback inhibition, increasing the hepatic conversion of cholesterol to bile acids. Decreasing concentrations of hepatic cholesterol causes a number of compensatory effects, such as increased plasma LDL and IDL uptake by increasing the number of high affinity receptors for LDLs present on cell membranes, especially in the liver, and induction of the HMG-CoA reductase enzyme. Despite the fact that resins cause increased hepatic cholesterol synthesis, there is a lowering of cholesterol levels in plasma. The selectivity of these compounds depends on the fact that the resins, positively charged, do not bind to all anions as well. For example, cholestyramine ions can only be displaced by other anions that have affinity for functional groups positively charged by resins (such as bile acids). Furthermore, resins can affect TGs and induce a 5% of increase in HDL-C levels [35].

Cholestyramine and colestipol are two hygroscopic molecules with a high molecular weight (>1,000,000). They are effective in lowering total and LDL-C, also reducing mortality and cardiovascular events (7% and 24%, respectively) [35]. However, they are usually not well tolerated since they show significant gastrointestinal side effects, including abdominal pain, heartburn, bloating, and constipation. Cholestyramine and colestipol are indicated in patients who do not respond adequately to dietary adjustments for the treatment of primary hypercholesterolemia—hypercholesterolemia associated with hypertriglyceridemia when the first represents the major therapeutic problem [36]. They are not indicated if the primary alteration is only hypertriglyceridemia. Cholestyramine and colestipol are not effective in patients with homozygous familial hypercholesterolemia who do not have functional receptors, as it does not alter the removal of plasma LDLs with non-receptor mechanisms, while they may be useful in heterozygous patients for receptor alteration. The recommended starting doses for adults range from 4 to 10 g a day before meals. A more pronounced effect can be achieved at maximal recommended doses of 24 and 30 g/day, for cholestyramine and colestipol respectively. The recommended dosage for patients aged 10–16 years is 4 g up to 10 g [37] . 

Colesevelam has six-fold higher bile acid-binding capacity and fewer side effects than cholestyramine, probably due to its greater binding affinity for glycocholic acid [38]. Studies reported that the maximum lipid-lowering effect of colesevelam was produced within 2 weeks of treatment, and then maintained during long-term therapy with this drug [39]. The recommended dosage is 3.75 g (one packet) once daily, or 1.875 g (one packet) twice daily.

The chronic use of resins can interfere with digestion, fat absorption, and liposoluble vitamins (Vitamin A, D, K, K1), the latter causing an increase in bleeding tendency due to hypoprothrombinemia from Vitamin K deficiency [36]. After a long period of administration, a reduction in serum or erythrocyte folate was also observed. Bile acid sequestrants may delay or reduce the absorption of certain drugs such as phenylbutazone, warfarin, chlorothiazide, tetracycline, penicillin G, phenobarbital, preparations of thyroid hormone and thyroxine, and digital. In addition, it may interfere with drugs (e.g., estrogens) that, like bile acids, are subject to enterohepatic circulation. It is advisable to administer any other medication at least one hour before or 4–6 h after taking resins [36].

### 2.4. Ezetimibe

Ezetimibe is the first representative of a group of drugs capable of selectively inhibiting the intestinal absorption of phytosterols and dietary cholesterol. Once orally taken, it is located on the small intestine brush lining and inhibits cholesterol absorption, resulting in a decrease in intestinal cholesterol passage to the liver (Figure 1) [40]. The ezetimibe molecular target is a sterol transport , Niemann-Pick C1-Like 1 (NPC1L1), responsible for intestinal cholesterol capture and absorption of phytosterols. Lower cholesterol absorption leads to increased receptor-mediated LDL uptake but, if the drug is used in monotherapy, lower cholesterol absorption may be offset by increased biosynthesis. The molecule appears to be selective because it does not interfere with the absorption of triglycerides, liposoluble vitamins, fatty acids, bile acids, progesterone, and ethinyl estradiol [41].

Following a single administration of 20 mg ezetimibe to fasted subjects, it was rapidly absorbed and extensively metabolized by glucuronidation (Cmax 2–3 h). Ezetimibe is excreted mostly in the feces, with a minor part appearing in the urine [41].

Ezetimibe is indicated both inassociation with statins, to reduce total cholesterol, LDL-C, and apoB in patients with primary hypercholesterolemia (family or non-familial heterozygosity) that are not adequately controlled with statins alone [42], as well as in monotherapy in these same patients for whom statins are considered inappropriate or not tolerated. The administration of ezetimibe 10 mg + simvastatin 10 mg reduces LDL-C serum levels (−44%) to the same extent as simvastatin 80 mg monotherapy [42]. It is also indicated for homozygous familial hypercholesterolemia in combination with atorvastatin or simvastatin, and homozygous familial sitosterolemia. In all cases, patient should not have responded to diet, physical activity, and other non-pharmacological measures [40].

The recommended dose for adults is one tablet of ezetimibe 10 mg once daily, while for children the start of treatment should be under the supervision of a specialist [41].

Alkaline and magnesium-based antacids reduce the Cmax of ezetimibe; cholestyramine may cause lower bioavailability of ezetimibe in cases of close administration (must be at least 2 h before or at least 4 h after administration of bile acid sequestrants); association with fibrates is not recommended as these increase the concentration of ezetimibe with a possible risk of cholelithiasis; cyclosporine may lead to increased concentrations of ezetimibe [40]. There are no significant interactions with warfarin or digoxin. It can be given independently of meals as these have no effect on its oral bioavailability.

### 2.5. Niacin

Niacin, also called vitamin B3, PP, or nicotinic acid, significantly raises HDL levels while decreasing those of VLDL and LDL with mechanisms that do not involve cholesterol biosynthesis or catabolism. This molecule, in fact, prevents lipolysis in adipose tissue as it is a powerful inhibitor of the intracellular lipase system, generating multiple effects that eventually lead to the reduction of plasma cholesterol and triglycerides [43]. Reduction of lipolysis decreases FFA mobilization, decreasing their levels in the liver, resulting in a decrease in the hepatic synthesis of triglycerides with consequent lower production of VLDL (Figure 1). A further mechanism of action of niacin consists of the ability of nicotinic acid to stimulate the activity of lipoprotein lipase, thus increasing the clearance of VLDL: the lower quantity of VLDL leads to reduced levels of LDL, which is derived from LDL (Figure 1) [43].

After oral administration, niacin is absorbed rapidly so that maximal plasma concentrations are reached in 30–60 min. The plasma half-life after administration of 1 g nicotinic acid is around 1 h [44].

Niacin is approved for the treatment of hypercholesterolemia and hypertriglyceridemia and, in both cases, daily oral doses of 1.5–3.5 g are generally sufficient. It is also indicated for familial combined hyperlipidaemia in patients who do not respond to non-pharmacological approaches such as diet and exercise. In addition, for the treatment of heterozygous familial hypercholesterolemia, 2–6 g is usually given per day. Niacin should be taken in multiple doses during the day, starting from 50–100 mg 2–3 times a day, and gradually increasing the dose whilst carefully checking for any side effects. The reduction of triglycerides can be already observed several hours after the intake of niacin, while the effects on cholesterol decrease take a few days.

The most common side effect of niacin is skin vasodilatation (flushing and itching), an annoying but harmless sensation that can be prevented by taking aspirin, ibuprofen, or indomethacin before the drug [43]. Extended-release niacin was tested in combination with laropiprant, an antagonist of the prostaglandin D2 receptor DP1 that has been shown to reduce flushing in up to two-thirds of patients, but was withdrawn due to increased risk of non-fatal, but serious side effects after preliminary data from a large, long-term study (HPS2-THRIVE) [45]. Gastrointestinal intolerance is an effect that can be minimized by administering the drug with meals [43]. Niacin is contraindicated in patients with liver disease, as it often causes a reversible increase in plasma levels of AST, ALT, LDH, and alkaline phosphatase, and should be used with caution in patients with diabetes mellitus and gout, given its ability to raise glucose and uric acid levels [44]. The association of niacin with HMG-CoA reductase inhibitors may lead to an increased risk of myopathy and rhabdomyolysis, whereas with bile acid sequestrants, it may reduce the bioavailability of niacin if the administrations are not spaced [46]. It is advisable to avoid the simultaneous use of niacin with adrenergic blockers and vasodilators (calcium channel blockers, nitrates) as there is a greater risk of vasodilation and postural hypotension.

### 2.6. Omega-3 Fatty Acids

Omega-3 fatty acids are polyunsaturated fatty acids with a double bond at the third carbon atom from the end of the carbon chain. They become part of the cell membrane, as with other fatty acids, and thanks to their chemical–physical characteristics, they determine the fluidity characteristics of membranes.

Omega-3 has shown to decrease CVD events as monotherapy in secondary prevention [47]. However, conflicting results are reported. Meta-analyses of omega-3 fatty acids added to optimal statin therapy suggest they give no added benefit [48]. The mechanisms by which omega-3 polyunsaturated fatty acids exert cardiovascular protective effects are both functional and metabolic: they cause greater fluidity of membranes, improve endothelial function, modulate platelet aggregation, modulate the metabolism of eicosanoids, and stabilize atheromatous lesions [47]. From a metabolic point of view, omega-3 mainly reduces serum triglycerides through an increase in the oxidation of fatty acids, further decreasing their synthesis and modulating the composition of membrane phospholipids. In addition, omega-3 increases LDL diameter (a characteristic that would therefore reduce its atherogenicity) without reducing its plasma levels [47].

Highly-purified omega-3 is usually used together with other drugs for the treatment of certain forms of hypertriglyceridemia [49]. The pharmaceutical form is represented by soft capsules of 1000 mg, containing 460–465 mg of eicosapentaenoic acid (EPA) and 375–380 mg of docosaexaenoic acid (DHA), taken twice a day. The common side effects are: stomach problems, indigestion (dyspepsia), and nausea [49]. Uncommon side-effects are abdominal and stomach pain, allergic reactions, dizziness, problems with taste, diarrhea, and vomiting. The use of omega 3 is not contraindicated in children and adolescents.

### 2.7. PCSK9 Inhibitors

#### 2.7.1. Alirocumab

Alirocumab is a fully human IgG1 monoclonal antibody that binds with high affinity and specificity to proprotein convertase subtilisin/kexin type 9 (PCSK9) by inhibiting it. PCSK9 usually binds to low density lipoprotein receptors (LDLR) on the surface of hepatocytes and is able to promote degradation of such receptors within the liver [50]. LDLRs are the main receptor that eliminates circulating LDLs, therefore, the reduction of LDLR levels by PCSK9 results in higher levels of circulating LDL-C (Figure 1). Alirocumab inhibits PCSK9 binding with LDLRs, thus increasing the number of LDLRs available to eliminate LDLs, thus lowering LDL-C levels. LDLR receptors also bind residues of VLDL rich in triglycerides and intermediate density lipoproteins (IDL). Therefore, treatment with alirocumab may result in a reduction in these lipoprotein residues, demonstrated by decreases in apolipoprotein B (Apo B), non-high density lipoprotein cholesterol (non-HDL-C), and triglycerides (TG) [51].

Alirocumab is indicated in adults with primary hypercholesterolemia (heterozygous or unhealthy family), in mixed dyslipidemia in addition to dietary changes, in combination with a statin or other hypolipidemic therapy in patients unable to reach LDL-C targets with the maximum tolerated dose of statin, or in monotherapy or combination therapy with other hypolipidemic therapies in statin-intolerant patients or patients for whom a statin is contraindicated [52]. 

Alirocumab has near 90% bioavailability and a long half-life (around 17–20 days) [53]. The initial dose of 75 mg is administered subcutaneously once every two weeks. In patients requiring a major reduction in LDL-C (>60%), an initial dose of 150 mg, always subcutaneously, may be given once every two weeks [54]. The dose of alirocumab can be personalized according to the patient’s characteristics, such as baseline LDL-C, target therapy, and response. Lipid levels can be evaluated four weeks after the start of treatment or after modification when a constant level of LDL-C is reached and the dose can be adjusted (increased or reduced) accordingly.

It is well known that statins and other therapies that modify the lipid profile increase the production of PCSK9, the protein on which alirocumab acts [51]. This results in an increase in target clearance and reduced systemic exposure to alirocumab. Compared to alirocumab monotherapy, alirocumab exposure is reduced by about 40% if the drug is used in combination with a statin, and about 15% if the drug is used in association with ezetimibe [51].

#### 2.7.2. Evolocumab

Evolocumab is an IgG2 human monoclonal antibody produced in Chinese hamster ovary cells (CHO) by recombinant DNA technology [51]. Evolocumab selectively binds PCSK9 and prevents its binding with the LDLR (Figure 1). Evolocumab results in a reduction in circulating PCSK9, LDL-C, total cholesterol (TC), ApoB, and non-HDL cholesterol, and an increase in HDL-C and Apo11 in patients with primary hypercholesterolemia and mixed dyslipidemia [55,56].

Evolocumab is indicated in adult patients with primary hypercholesterolemia (familial heterozygous and non-familial) or mixed dyslipidemia, in addition dietary changes. It can be used in combination with a statin, with statins and other hypolipidemic therapies in patients who do not reach target levels of LDL-C with a maximum tolerated statin dose, or in monotherapy or in combination with other hypolipidemic therapies in statin-intolerant patients or patients for whom the use of statins is contraindicated [57].

Evolocumab has 70% bioavailability and a long half-life (around 11–17 days) [53]. The recommended adult dose of evolocumab is 140 mg every two weeks or 420 mg once a month for primary hypercholesterolemia and mixed dyslipidaemia; the two doses are clinically equivalent.

For homozygous familial hypercholesterolemia in adults and adolescents aged 12 years or over, the recommended starting dose is 420 mg once a month. After 12 weeks of treatment and no clinically-relevant response, it is possible to increase the frequency of administration to 420 mg every two weeks [57]. 

No formal drug–drug interaction studies have been conducted for evolocumab. The pharmacokinetic interactions between statins and evolocumab have been studied. In statin-associated patients, an increase in evolocumab clearance of approximately 20% was observed, partly due to increased concentrations of statin-induced PCSK9. No statin dose adjustments are required when used in association with evolocumab [51]. 

### 2.8. Lomitapide

Lomitapide represents a new therapeutic approach for patients with homozygous FH who, despite the widespread use of statins, do not reach LDL targets. It is not authorized for heterozygous FH. It is a selective inhibitor of the microsomal transport protein of triglycerides (MTP), an intracellular protein found in the endoplasmic reticulum of liver and intestine cells which plays a role in the assembly of fats, such as cholesterol and triglycerides, in lipoproteins, and their subsequent release into the blood. The inhibition of MTP reduces the production of chylomicrons in enterocytes and increases production of very low-density lipoprotein (VLDL) cholesterol in hepatocytes independently of the LDL receptor (Figure 1) [58].

The absolute oral bioavailability of lomitapide is 7% due to an extensive first pass effect. Doses higher than 60 mg suggest a trend toward non-linearity [59].

The efficacy of lomitapide, at a starting dose of 5 mg/day for the first two weeks and then escalated to 10, 20, 40, and 60 mg/day at 4-week intervals, was evaluated in a single-arm open-label study of 29 patients with homozygous familial hypercholesterolemia, in addition to their current lipid-lowering therapy (statins, ezetimibe, and apheresis) [60]. At week 26, the reduction in plasma LDL cholesterol levels was approximately 40% when compared to baseline values (*p* < 0.001). Furthermore, serum triglyceride levels were reduced by 31%, whereas HDL-C and Lp(a) levels showed only transient decreases [60].

Lomitapide should be used in combination with a low-fat diet and other lipid-lowering drugs, administered on an empty stomach at least two hours after the evening meal, as fat content can adversely affect gastrointestinal tolerability. The starting dose is 5 mg/day, and after several weeks it is possible to gradually increase the dose up to the maximum recommended dose of 60 mg [59].

The most common, least-serious side effects are those affecting the gastrointestinal tract (diarrhea, nausea, dyspepsia, vomiting) with an incidence of about 90%, while the most serious side effects are those affecting the liver, such as abnormal increases in liver transaminases and hepatic steatosis [60]. The use of CYP3A4 inhibitors should be avoided when patients are treated with lomitapide, otherwise it is necessary to suspend therapy with the latter [61]. CYP3A4 inducers may reduce the effect of the drug. Patients taking warfarin should have their INR monitored regularly, as lomitapide increases plasma warfarin concentrations with the risk of causing over-therapeutic anticoagulation [62]. Finally, the plasma concentrations of statins can also be increased by lomitapide such that constant monitoring of this association needs to be performed since it may lead to a greater risk of myopathy [61].

### 2.9. Mipomersen

Mipomersen is an antisense oligonucleotide that inhibits the production of apoB-100 by binding to the mRNA that encodes the synthesis of apoB, an essential component of VLDL and LDL [63]. Mipomersen reduces the liver levels of mRNA for apoB-100 in a dose-dependent manner, followed by a reduction in LDL-C, LDL, TG, and lipoprotein(a).

Mipomersen half-life is approximately two to five hours, and it has an elimination half-life of one to two months [64]. It is more than 90% protein-bound and it is metabolized by tissue endonucleases. The estimated subcutaneous bioavailability of mipomersen is between 54% and 78% after a once-weekly dose of 50 to 400 mg [64].

The lipid-lowering effects of mipomersen in two phase three clinical trials have been shown after failed courses of standard lipid-lowering therapy [65]. 

A phase 3 clinical trial in 158 patients with hypercholesterolemia (LDL-C > 100 mg/dL) taking maximally tolerated doses of statins and once-weekly 200 mg subcutaneous mipomersen for a 26-week treatment course showed an average LDL-C reduction of 37% from baseline [65]. Interestingly, half of the mipomersen-treated patients were able to reach the goal (LDL-C levels < 70 mg/dL). However, the protective effects of mipomersen on cardiovascular outcomes needs to be better evaluated.

Mipomersen is indicated as an adjunct to lipid-lowering drugs to reduce LDL-C, Apo B, TG, and non-HDL-C in patients with homozygous FH, and in particular in patients that are resistant to current standards of care. The use of the drug has already been approved in the United States by the FDA for HoFH, while the EMA has expressed negative opinions on the approval of the drug, mainly because of its side effects [66]. The recommended dose is 200 mg/mL, subcutaneously, once weekly, on the same day each week.

Two hepatic side-effects of mipomersen have been reported: elevation of plasma alanine aminotransferase (ALT) levels and hepatic fat accumulation, an effect of mipomersen that is related to its mode of action [66]. Longer-term evaluations of transaminase elevations and hepatic steatosis are necessary.

Mipomersen is not recommended for patients with severe renal dysfunction and/or liver dysfunction. Common adverse effects reported in clinical trials include injection site reactions and flu-like symptoms [65]. Injection site reactions occurred three-times more often in the treatment group than the placebo group. Most of these reactions were described as mild, erythematous, and painless, and resolved after 24 h. Flu-like symptoms, the second most common complaint that arises frequently in the first doses, appeared shortly after the administration of mipomersen and resolved within a few days.

## 3. Discussion

To date, statins remain the first choice therapy as they have been shown to reduce the annual risk of major vascular events by 21% [15] and the annual risk of major coronary events by 24% through lowering of LDL-C by 40 mg/dl [67]. The expected reduction in LDL-C depends on the statin used, and is approximately 30–50% for a statin with moderate power and >50% for a high-power statin [68]. Furthermore, ezetimibe represents the second-choice drug to be used in combination with statins if the lipid target cannot be reached in high and very high cardiovascular risk patients [15]. In the IMPROVE-IT study conducted on 18,444 patients with acute coronary syndrome, ezetimibe in association with simvastatin demonstrated an additional reduction in LDL-C levels of 17% when compared with simvastatin alone, highlighting a reduction, at 7 years, of the absolute risk of death from cardiovascular diseases, major coronary events, and non-fatal stroke of 2% (HR 0.936; IC 95%, 0.89–0.99, *p* = 0.016) [69].

Other potential strategies include fibrates, bile acid sequestrants, and niacin [19]. Fibrates may be more useful if added to a statin in patients with low HDL-C and high TGs, even if this needs to be confirmed in a large clinical trial. In addition, due to tolerability and safety concerns, combination therapy with a statin needs to be evaluated and in some cases discouraged, like for gemfibrozil, due to the increased risk of myopathy and hepatotoxicity. Modest efficacy and problems with tolerability and compliance have been responsible for relegating traditional bile acid sequestrants, such as cholestyramine and colestipol, to an adjunctive role. The newest bile resin colesevelam can be useful in those patients who are not able to tolerate first-line therapy. Regarding niacin, end-point studies of this drug combined with statins have been disappointing, and considerations, especially for benefit/risk assessment, need to be evaluated for side effects.

However, recent studies demonstrated the effects of two new monoclonal antibodies in decreasing LDL-C values. In particular, the FOURIER and ODYSSEY studies reported the results of the PCSK9 inhibitors evolocumab and alirocumab, respectively [70,71]. While results regarding the effectiveness in reducing cardiovascular events of the ODYSSEY study will be available later this year, data obtained by FOURIER trial on cardiovascular outcomes (the primary endpoint was the composite of cardiovascular death, myocardial infarction, stroke, coronary revascularisation, or unstable angina; the key secondary endpoint was the composite of cardiovascular death, myocardial infarction, or stroke) were recently published [56]. The authors reported that there was a highly significant monotonic relationship between low LDL-C levels and a lower risk of the primary and secondary efficacy composite endpoints (LDL-C concentrations of less than 0.2 mmol/L; *p* = 0.0012 for the primary endpoint, *p* = 0.0001 for the secondary endpoint) [56]. Nevertheless, according to National Lipid Association, new monoclonal antibodies should only be reserved for heterozygous hypercholesterolemic adult patients with high or very high cardiovascular risk, or for non-familial hypercholesterolemic patients with high cardiovascular risk and high LDL-C values which differ by at least 30% from the target value despite proper use of statins and ezetimibe [72]. Pending the publication of results on the reduction of cardiovascular events associated with the use of the PCSK9 inhibitor alirocumab, a minimum deviation of 30% compared to the lipid target can be used to identify the patients who will most benefit from the absolute reduction of LDL cholesterol by the new therapeutic strategies. It is recognized that the same doses of the same statin may produce different effects in terms of decreasing LDL-C, and the same levels of LDL-C can be achieved with different treatment periods [73]. These differences appear to be attributable to genetic polymorphisms with still unclear mechanisms [74]. Treatment should therefore be carried out at the maximum-tolerated dose of statin by verifying adherence and time of intake, and also by optimizing diet and lifestyle [15]. Furthermore, a systematic review of lipid-lowering therapies added to medium- to high-intensity statin therapy confirmed the substantial LDL-C reductions of PCSK9 inhibitors versus placebo or ezetimibe in individual trials. Among the PCSK9 inhibitors, evolocumab appeared to have a greater reduction in LDL-C levels than alirocumab (75 mg biweekly, ~20%; 150 mg biweekly, ~10%; 300 mg monthly, ~20%) [75]. 

Other currently available lipid-lowering agents for the management of FH are mipomersen, an antisense single-strand oligonucleotide that inhibits the production of apoB, and Lomitapide, which inhibits the enzyme that transfers triglycerides onto apoB. However, these specialized therapies may be needed to control LDL-C in patients with atherosclerotic cardiovascular disease risk, baseline LDL-C > 190 mg/dL, and/or phenotypic HoFH, who have an inadequate response to statins with or without ezetimibe and PCSK9 inhibitors [58]. Mipomersen and lomitapide are only indicated for HoFH and may be prescribed at the discretion of lipid specialists. As the mechanisms of action of these novel agents do not involve upregulation of the LDL receptor, these drugs may be of particular benefit in LDL receptor-negative HoFH patients. However, the effect of mipomersen and lomitapide on cardiovascular morbidity and mortality needs to be better determined.

We have to take in consideration that, from analysis of the literature, 83,858 patients treated with five different statins accounted for only 0.11% of patients who accused myositis and 0.016% who accused rhabdomyolysis [76]. Regarding myalgia, unlike myositis and rhabdomyolysis, clinical practice has an incidence ranging from five to between 10 and 15% [76]. The risk of myopathy can be minimized by detecting and monitoring the most vulnerable patients, and by avoiding possible drug interactions due to the concomitant administration of medicines that inhibit transport proteins (OATP, P-gp) and some CYP450 isoenzymes (such as cyclosporin, tacrolimus, macrolides, azole antimycotics, some calcium antagonists, and HIV protease inhibitors).

Numerous adverse events associated with the use of statins are frequently dependent on the type of active principle administered. Many patients may be intolerant to some statins, although they are not intolerant to others. Intolerance to statin use should therefore take into account various parameters such as symptomatology, CPK level, and the effects caused by the switch to different statins [76]. However, only after the third attempt can the patient be considered intolerant to statin treatment.

Another problem affecting statins is the adherence to therapy. In fact, it is known from the literature that approximately 50% of patients treated with statins are not adherent to therapy. Adherence to therapy has a very important impact in terms of decreasing the risk of cardiovascular events (RR 0.85, IC 95%: 0.81–0.89) and mortality from any cause (RR 0.55, IC 95%: 0.46–0.67) [77]. Adherence should therefore be assured by the patient and monitored by the physician (specialist in collaboration with the general practitioner) based on the control of LDL-C values.

## 4. Conclusions

In recent decades, numerous clinical trials have demonstrated the efficacy of several drug treatments able to reduce the incidence of new atherothrombotic episodes in survivors of a first episode. Statins, in particular, have been able to reduce cardiovascular mortality and major non-fatal atherothrombotic events in heterogeneous populations through both primary and secondary prevention. These treatments are part of the recommendations of both US and European guidelines, and should be prescribed to all patients who have already had a cardiovascular event and have no specific contraindication. For anti-PCSK9 monoclonal antibodies, conversely, the cost–effectiveness debate has just begun. Although it is possible to achieve with these drugs a very important therapeutic goal, in view of the costs for new therapy in the United States of between $14,100/year and $14,600/year [78], some doubts have also arisen on the basis of the vast potential of users, especially when compared to classical statin therapy ($100–180/year). This problem becomes more evident for mipomersen and lomitapide since one year of treatment costs more than $170,000/year and $270,000/year, respectively, such that cost and concern for tolerability will remain limiting factors for these two agents [79].
